# Correction to: Context dependency of nucleotide probabilities and variants in human DNA

**DOI:** 10.1186/s12864-022-08490-z

**Published:** 2022-05-10

**Authors:** Yuhu Liang, Christian Grønbæk, Piero Fariselli, Anders Krogh

**Affiliations:** 1 Department of Computer Science, University of Copenhagen, Copenhagen, Denmark; 2 Department of Biology, University of Copenhagen, Copenhagen, Denmark; 3 Present address: Novo Nordisk Foundation Center for Basic Metabolic Research, University of Copenhagen, Copenhagen, Denmark; 4 Department of Medical Sciences, University of Torino, Torino, Italy; 5 Center for Health Data Science, University of Copenhagen, Copenhagen, Denmark

**Correction to: BMC Genomics 23, 87 (2022)**

**https://doi.org/10.1186/s12864-021-08246-1**

Following publication of the original article [1], it was reported that Christian Grønbæk was missing affiliation 2 in the authorship panel. The correct authorship list is given in this Correction article and the original article [1] has been updated.
