# Supplementary material for: Context dependency of nucleotide probabilities and variants in human DNA
Source: BMC Genomics. 2022 Jan 31;23:87. doi: 10.1186/s12864-021-08246-1 (PMC8802520; doi:10.1186/s12864-021-08246-1)
Supplement: Supplementary file 2 — Additional file 2 Supplementary figures: Figure S1, S2, S3, S4, S5. [file 12864_2021_8246_MOESM2_ESM.pdf]

Supplementary Figures for

Context dependency of nucleotide probabilities and variants in human DNA

Yuhu Liang[1,†], Christian Grønbæk[2,†], Piero Fariselli[3] and Anders Krogh[1,4,*,†]

[1]Department of Computer Science, University of Copenhagen, Denmark
[2]Novo Nordisk Foundation Center for Basic Metabolic Research, University of Copenhagen, Denmark
[3]Department of Medical Sciences, University of Torino, Italy
[4]Center for Health Data Science, University of Copenhagen, Denmark
[†]Part of this work was carried out at Department of Biology, University of Copenhagen, Denmark

Corresponding author: Anders Krogh, email: akrogh@di.ku.dk

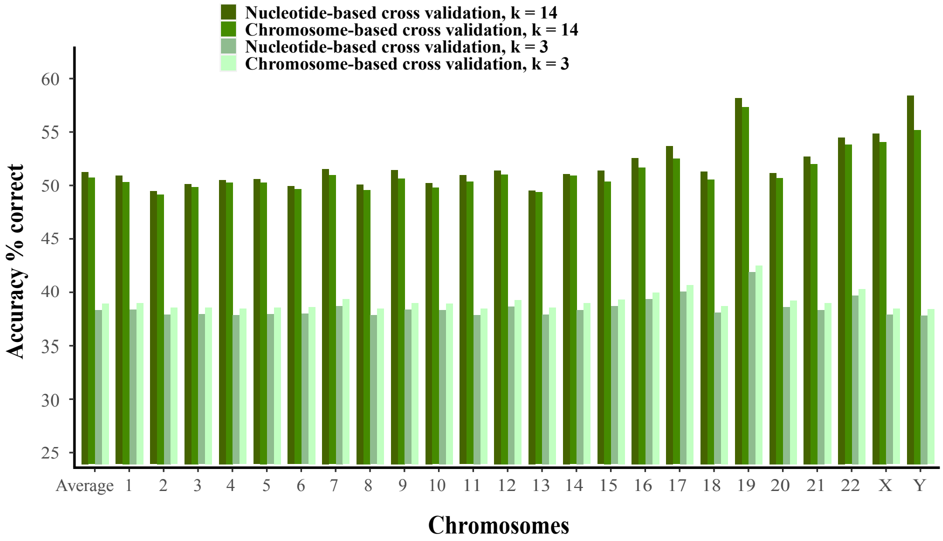

Figure S1: Chromosome based cross validations for baseline model and Bidir-Markov model. For each chromosome, the overall prediction accuracy is calculated for a model is estimated from the other chromosomes (chromosome-based cross validation). The overall average is weighted by chromosome sizes. These are compared to the nucleotide-based cross validation accuracies used in Figure 1.

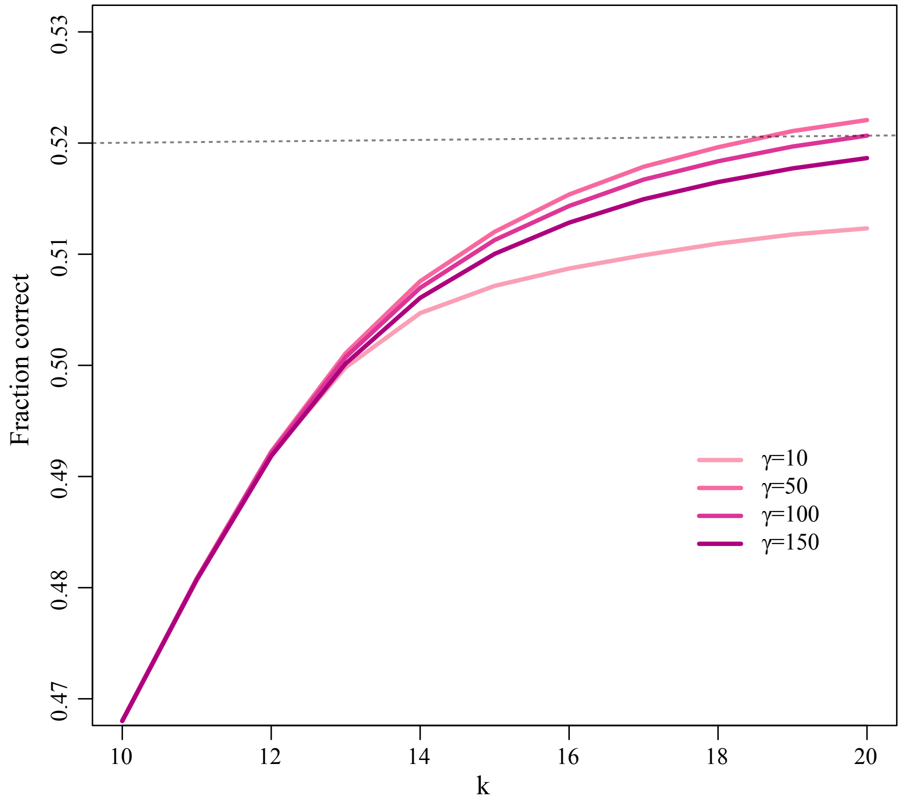

Figure S2: Accuracy of the bi-directional k-th order Markov model for different strengths of regularization, γ. Results are shown only for Chromosome 20 with the model estimated from all the other chromosomes.

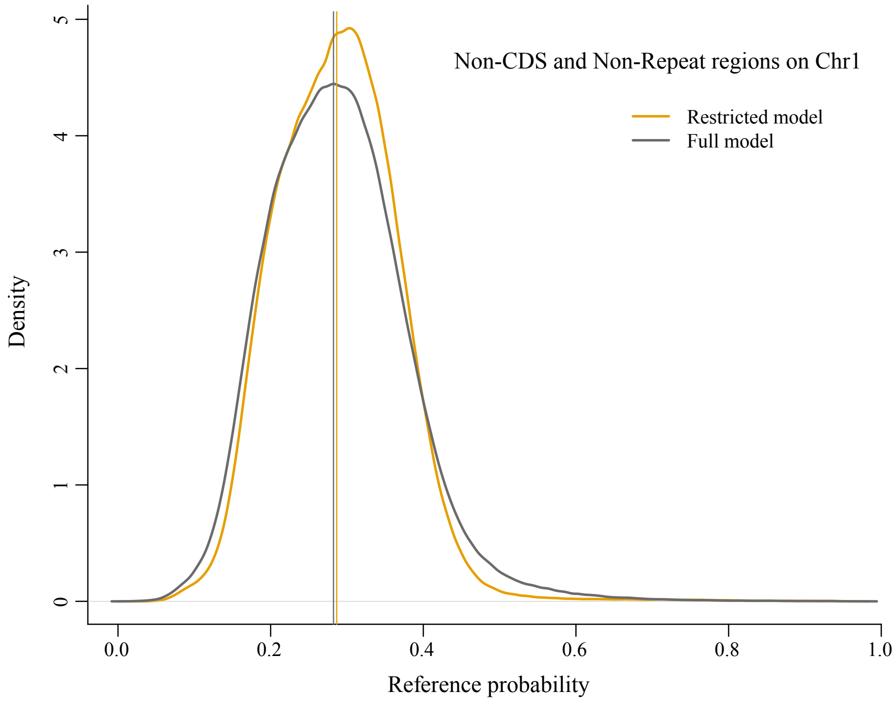

Figure S3: Comparison of restricted and full model based on density profile of reference probabilities. Density profile of the reference probabilities for the full model was shown as a dark grey line and the other for a model estimated on non-repeat and non-coding regions on Chromosome 1. The yellow and gray vertical lines represent the median probabilities of restricted model and full model, which are 0.286578 and 0.282368, respectively.

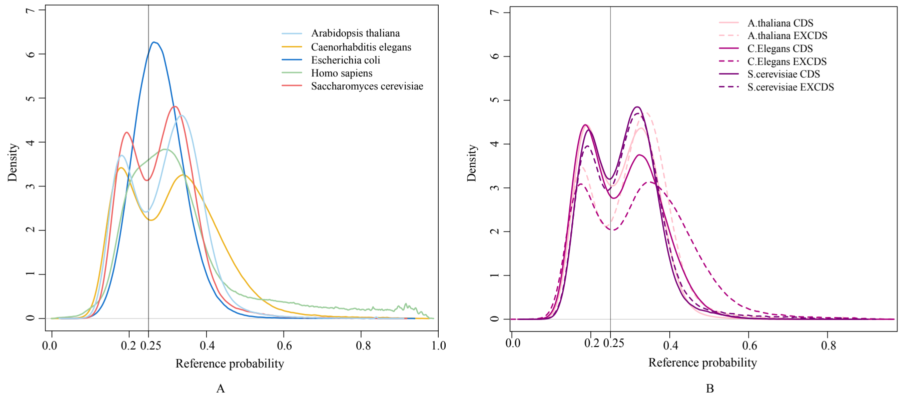

Figure S4: Density profile of reference probabilities of different species. A. Those species were estimated via 10-k context bidirectional Markov model, γ = 100 interpolated from 6. B. Density plots of CDS regions and non-CDS for the species, which have two peaks in Figure S4A.

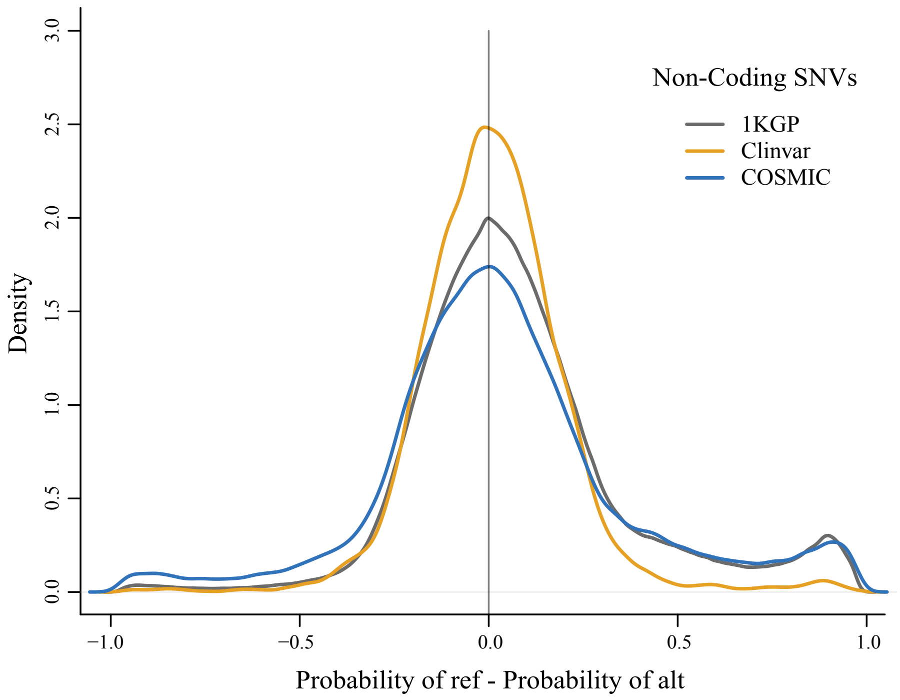

Figure S5: Density profiles of Pref - Palt for SNPs on Chromosome 1. Density profiles show ClinVar, somatic mutations (COSMIC) and 1KGP SNPs in Non-Coding regions, respectively.
